# Supplementary material for: A protocol for the VISION study: An indiVidual patient data meta-analysis of randomised trials comparing MRI-targeted biopsy to standard transrectal ultraSound guided bIopsy in the detection of prOstate cancer
Source: PLoS One. 2022 Feb 3;17(2):e0263345. doi: 10.1371/journal.pone.0263345 (PMC8812968; doi:10.1371/journal.pone.0263345)

**Supplementary Appendix to “A protocol for the VISION study: An indiVidual patient data meta-analysis of randomised trials comparing MRI-targeted biopsy to standard transrectal ultraSound guided bIopsy in the detection of prOstate cancer”**

**Authors:** Veeru Kasivisvanathan, Vinson Wai-Shun Chan, Keiran D Clement, Brooke Levis, Masoom Haider, Ridhi Agarwal, Jonathan Deeks, Mark Emberton, Gregory R Pond, Yemisi Takwoingi, Laurence Klotz**^^^**, Caroline M Moore**^^^**, VISION study collaborators

^These authors share joint senior authorship

Contents

[PRISMA-P (Preferred Reporting Items for Systematic review and Meta-Analysis Protocols) 2015 checklist 2](#_Toc78716953)

[Preferred Reporting Items for Systematic reviews and Meta-Analyses (PRISMA) statement 4](#_Toc78716954)

[The Preferred Reporting Items for Systematic reviews and Meta-Analyses (PRISMA) statement – individual patient data extension 7](#_Toc78716955)

[The Preferred Reporting Items for Systematic reviews and Meta-Analyses (PRISMA) statement – Diagnostic Test Accuracy Extension 11](#_Toc78716956)

[Search Strategy 14](#_Toc78716957)

[Anticipated Codebook 15](#_Toc78716958)

[QUADAS-C Tool 32](#_Toc78716959)

[RoB 2.0 tool 38](#_Toc78716960)

[Domain 3: Risk of bias due to missing outcome data 53](#_Toc78716961)

[Domain 4: Risk of bias in measurement of the outcome 56](#_Toc78716962)

[Domain 5: Risk of bias in selection of the reported result 59](#_Toc78716963)

[Standards of Reporting for MRI-targeted Biopsy Studies (START) of the Prostate 67](#_Toc78716964)

[Intention-to-treat and modified intention-to-treat definitions and approaches 69](#_Toc78716965)

# PRISMA-P (Preferred Reporting Items for Systematic review and Meta-Analysis Protocols) 2015 checklist

**PRISMA-P (Preferred Reporting Items for Systematic review and Meta-Analysis Protocols) 2015 checklist: recommended items to address in a systematic review protocol***

| Section and topic | Item No | Checklist item |
| --- | --- | --- |
| ADMINISTRATIVE INFORMATION | | |
| Title: |  |  |
| Identification | 1a | Identify the report as a protocol of a systematic review |
| Update | 1b | If the protocol is for an update of a previous systematic review, identify as such |
| Registration | 2 | If registered, provide the name of the registry (such as PROSPERO) and registration number |
| Authors: |  |  |
| Contact | 3a | Provide name, institutional affiliation, e-mail address of all protocol authors; provide physical mailing address of corresponding author |
| Contributions | 3b | Describe contributions of protocol authors and identify the guarantor of the review |
| Amendments | 4 | If the protocol represents an amendment of a previously completed or published protocol, identify as such and list changes; otherwise, state plan for documenting important protocol amendments |
| Support: |  |  |
| Sources | 5a | Indicate sources of financial or other support for the review |
| Sponsor | 5b | Provide name for the review funder and/or sponsor |
| Role of sponsor or funder | 5c | Describe roles of funder(s), sponsor(s), and/or institution(s), if any, in developing the protocol |
| INTRODUCTION | | |
| Rationale | 6 | Describe the rationale for the review in the context of what is already known |
| Objectives | 7 | Provide an explicit statement of the question(s) the review will address with reference to participants, interventions, comparators, and outcomes (PICO) |
| METHODS | | |
| Eligibility criteria | 8 | Specify the study characteristics (such as PICO, study design, setting, time frame) and report characteristics (such as years considered, language, publication status) to be used as criteria for eligibility for the review |
| Information sources | 9 | Describe all intended information sources (such as electronic databases, contact with study authors, trial registers or other grey literature sources) with planned dates of coverage |
| Search strategy | 10 | Present draft of search strategy to be used for at least one electronic database, including planned limits, such that it could be repeated |
| Study records: |  |  |
| Data management | 11a | Describe the mechanism(s) that will be used to manage records and data throughout the review |
| Selection process | 11b | State the process that will be used for selecting studies (such as two independent reviewers) through each phase of the review (that is, screening, eligibility and inclusion in meta-analysis) |
| Data collection process | 11c | Describe planned method of extracting data from reports (such as piloting forms, done independently, in duplicate), any processes for obtaining and confirming data from investigators |
| Data items | 12 | List and define all variables for which data will be sought (such as PICO items, funding sources), any pre-planned data assumptions and simplifications |
| Outcomes and prioritization | 13 | List and define all outcomes for which data will be sought, including prioritization of main and additional outcomes, with rationale |
| Risk of bias in individual studies | 14 | Describe anticipated methods for assessing risk of bias of individual studies, including whether this will be done at the outcome or study level, or both; state how this information will be used in data synthesis |
| Data synthesis | 15a | Describe criteria under which study data will be quantitatively synthesised |
|  | 15b | If data are appropriate for quantitative synthesis, describe planned summary measures, methods of handling data and methods of combining data from studies, including any planned exploration of consistency (such as I^2^, Kendall’s τ) |
|  | 15c | Describe any proposed additional analyses (such as sensitivity or subgroup analyses, meta-regression) |
|  | 15d | If quantitative synthesis is not appropriate, describe the type of summary planned |
| Meta-bias(es) | 16 | Specify any planned assessment of meta-bias(es) (such as publication bias across studies, selective reporting within studies) |
| Confidence in cumulative evidence | 17 | Describe how the strength of the body of evidence will be assessed (such as GRADE) |

*** It is strongly recommended that this checklist be read in conjunction with the PRISMA-P Explanation and Elaboration (cite when available) for important clarification on the items. Amendments to a review protocol should be tracked and dated. The copyright for PRISMA-P (including checklist) is held by the PRISMA-P Group and is distributed under a Creative Commons Attribution Licence 4.0.**

*From: Shamseer L, Moher D, Clarke M, Ghersi D, Liberati A, Petticrew M, Shekelle P, Stewart L, PRISMA-P Group. Preferred reporting items for systematic review and meta-analysis protocols (PRISMA-P) 2015: elaboration and explanation. BMJ. 2015 Jan 2;349(jan02 1):g7647.*

# Preferred Reporting Items for Systematic reviews and Meta-Analyses (PRISMA) statement

| **Section and Topic** | **Item #** | **Checklist item** | **Location where item is reported** |
| --- | --- | --- | --- |
| **TITLE** | | |  |
| Title | 1 | Identify the report as a systematic review. |  |
| **ABSTRACT** | | |  |
| Abstract | 2 | See the PRISMA 2020 for Abstracts checklist. |  |
| **INTRODUCTION** | | |  |
| Rationale | 3 | Describe the rationale for the review in the context of existing knowledge. |  |
| Objectives | 4 | Provide an explicit statement of the objective(s) or question(s) the review addresses. |  |
| **METHODS** | | |  |
| Eligibility criteria | 5 | Specify the inclusion and exclusion criteria for the review and how studies were grouped for the syntheses. |  |
| Information sources | 6 | Specify all databases, registers, websites, organisations, reference lists and other sources searched or consulted to identify studies. Specify the date when each source was last searched or consulted. |  |
| Search strategy | 7 | Present the full search strategies for all databases, registers and websites, including any filters and limits used. |  |
| Selection process | 8 | Specify the methods used to decide whether a study met the inclusion criteria of the review, including how many reviewers screened each record and each report retrieved, whether they worked independently, and if applicable, details of automation tools used in the process. |  |
| Data collection process | 9 | Specify the methods used to collect data from reports, including how many reviewers collected data from each report, whether they worked independently, any processes for obtaining or confirming data from study investigators, and if applicable, details of automation tools used in the process. |  |
| Data items | 10a | List and define all outcomes for which data were sought. Specify whether all results that were compatible with each outcome domain in each study were sought (e.g. for all measures, time points, analyses), and if not, the methods used to decide which results to collect. |  |
|  | 10b | List and define all other variables for which data were sought (e.g. participant and intervention characteristics, funding sources). Describe any assumptions made about any missing or unclear information. |  |
| Study risk of bias assessment | 11 | Specify the methods used to assess risk of bias in the included studies, including details of the tool(s) used, how many reviewers assessed each study and whether they worked independently, and if applicable, details of automation tools used in the process. |  |
| Effect measures | 12 | Specify for each outcome the effect measure(s) (e.g. risk ratio, mean difference) used in the synthesis or presentation of results. |  |
| Synthesis methods | 13a | Describe the processes used to decide which studies were eligible for each synthesis (e.g. tabulating the study intervention characteristics and comparing against the planned groups for each synthesis (item #5)). |  |
|  | 13b | Describe any methods required to prepare the data for presentation or synthesis, such as handling of missing summary statistics, or data conversions. |  |
|  | 13c | Describe any methods used to tabulate or visually display results of individual studies and syntheses. |  |
|  | 13d | Describe any methods used to synthesize results and provide a rationale for the choice(s). If meta-analysis was performed, describe the model(s), method(s) to identify the presence and extent of statistical heterogeneity, and software package(s) used. |  |
|  | 13e | Describe any methods used to explore possible causes of heterogeneity among study results (e.g. subgroup analysis, meta-regression). |  |
|  | 13f | Describe any sensitivity analyses conducted to assess robustness of the synthesized results. |  |
| Reporting bias assessment | 14 | Describe any methods used to assess risk of bias due to missing results in a synthesis (arising from reporting biases). |  |
| Certainty assessment | 15 | Describe any methods used to assess certainty (or confidence) in the body of evidence for an outcome. |  |
| **RESULTS** | | |  |
| Study selection | 16a | Describe the results of the search and selection process, from the number of records identified in the search to the number of studies included in the review, ideally using a flow diagram. |  |
|  | 16b | Cite studies that might appear to meet the inclusion criteria, but which were excluded, and explain why they were excluded. |  |
| Study characteristics | 17 | Cite each included study and present its characteristics. |  |
| Risk of bias in studies | 18 | Present assessments of risk of bias for each included study. |  |
| Results of individual studies | 19 | For all outcomes, present, for each study: (a) summary statistics for each group (where appropriate) and (b) an effect estimate and its precision (e.g. confidence/credible interval), ideally using structured tables or plots. |  |
| Results of syntheses | 20a | For each synthesis, briefly summarise the characteristics and risk of bias among contributing studies. |  |
|  | 20b | Present results of all statistical syntheses conducted. If meta-analysis was done, present for each the summary estimate and its precision (e.g. confidence/credible interval) and measures of statistical heterogeneity. If comparing groups, describe the direction of the effect. |  |
|  | 20c | Present results of all investigations of possible causes of heterogeneity among study results. |  |
|  | 20d | Present results of all sensitivity analyses conducted to assess the robustness of the synthesized results. |  |
| Reporting biases | 21 | Present assessments of risk of bias due to missing results (arising from reporting biases) for each synthesis assessed. |  |
| Certainty of evidence | 22 | Present assessments of certainty (or confidence) in the body of evidence for each outcome assessed. |  |
| **DISCUSSION** | | |  |
| Discussion | 23a | Provide a general interpretation of the results in the context of other evidence. |  |
|  | 23b | Discuss any limitations of the evidence included in the review. |  |
|  | 23c | Discuss any limitations of the review processes used. |  |
|  | 23d | Discuss implications of the results for practice, policy, and future research. |  |
| **OTHER INFORMATION** | | |  |
| Registration and protocol | 24a | Provide registration information for the review, including register name and registration number, or state that the review was not registered. |  |
|  | 24b | Indicate where the review protocol can be accessed, or state that a protocol was not prepared. |  |
|  | 24c | Describe and explain any amendments to information provided at registration or in the protocol. |  |
| Support | 25 | Describe sources of financial or non-financial support for the review, and the role of the funders or sponsors in the review. |  |
| Competing interests | 26 | Declare any competing interests of review authors. |  |
| Availability of data, code and other materials | 27 | Report which of the following are publicly available and where they can be found: template data collection forms; data extracted from included studies; data used for all analyses; analytic code; any other materials used in the review. |  |

*From:*  Page MJ, McKenzie JE, Bossuyt PM, Boutron I, Hoffmann TC, Mulrow CD, et al. The PRISMA 2020 statement: an updated guideline for reporting systematic reviews. BMJ 2021;372:n71. doi: 10.1136/bmj.n71

For more information, visit: <http://www.prisma-statement.org/>

# The Preferred Reporting Items for Systematic reviews and Meta-Analyses (PRISMA) statement – individual patient data extension

**PRISMA-IPD Checklist of items to include when reporting a systematic review and meta-analysis of individual participant data (IPD)**

| **PRISMA-IPD**  **Section/topic** | **Item No** | **Checklist item** | **Reported on page** |
| --- | --- | --- | --- |
| **Title** | | | |
| Title | 1 | Identify the report as a systematic review and meta-analysis of individual participant data. |  |
| **Abstract** | | | |
| Structured summary | 2 | Provide a structured summary including as applicable: |  |
|  |  | **Background**: state research question and main objectives, with information on participants, interventions, comparators and outcomes. |  |
|  |  | **Methods**: report eligibility criteria; data sources including dates of last bibliographic search or elicitation, noting that IPD were sought; methods of assessing risk of bias. |  |
|  |  | **Results**: provide number and type of studies and participants identified and number (%) obtained; summary effect estimates for main outcomes (benefits and harms) with confidence intervals and measures of statistical heterogeneity. Describe the direction and size of summary effects in terms meaningful to those who would put findings into practice. |  |
|  |  | **Discussion:** state main strengths and limitations of the evidence, general interpretation of the results and any important implications. |  |
|  |  | **Other:** report primary funding source, registration number and registry name for the systematic review and IPD meta-analysis. |  |
| **Introduction** | | | |
| Rationale | 3 | Describe the rationale for the review in the context of what is already known. |  |
| Objectives | 4 | Provide an explicit statement of the questions being addressed with reference, as applicable, to participants, interventions, comparisons, outcomes and study design (PICOS). Include any hypotheses that relate to particular types of participant-level subgroups. |  |
| **Methods** | | | |
| Protocol and registration | 5 | Indicate if a protocol exists and where it can be accessed. If available, provide registration information including registration number and registry name. Provide publication details, if applicable. |  |
| Eligibility criteria | 6 | Specify inclusion and exclusion criteria including those relating to participants, interventions, comparisons, outcomes, study design and characteristics (e.g. years when conducted, required minimum follow-up). Note whether these were applied at the study or individual level i.e. whether eligible participants were included (and ineligible participants excluded) from a study that included a wider population than specified by the review inclusion criteria. The rationale for criteria should be stated. |  |
| Identifying studies - information sources | 7 | Describe all methods of identifying published and unpublished studies including, as applicable: which bibliographic databases were searched with dates of coverage; details of any hand searching including of conference proceedings; use of study registers and agency or company databases; contact with the original research team and experts in the field; open adverts and surveys. Give the date of last search or elicitation. |  |
| Identifying studies - search | 8 | Present the full electronic search strategy for at least one database, including any limits used, such that it could be repeated. |  |
| Study selection processes | 9 | State the process for determining which studies were eligible for inclusion. |  |
| Data collection processes | 10 | Describe how IPD were requested, collected and managed, including any processes for querying and confirming data with investigators. If IPD were not sought from any eligible study, the reason for this should be stated (for each such study). |  |
|  |  | If applicable, describe how any studies for which IPD were not available were dealt with. This should include whether, how and what aggregate data were sought or extracted from study reports and publications (such as extracting data independently in duplicate) and any processes for obtaining and confirming these data with investigators. |  |
| Data items | 11 | Describe how the information and variables to be collected were chosen. List and define all study level and participant level data that were sought, including baseline and follow-up information. If applicable, describe methods of standardising or translating variables within the IPD datasets to ensure common scales or measurements across studies. |  |
| IPD integrity | A1 | Describe what aspects of IPD were subject to data checking (such as sequence generation, data consistency and completeness, baseline imbalance) and how this was done. |  |
| Risk of bias assessment in individual studies. | 12 | Describe methods used to assess risk of bias in the individual studies and whether this was applied separately for each outcome. If applicable, describe how findings of IPD checking were used to inform the assessment. Report if and how risk of bias assessment was used in any data synthesis. |  |
| Specification of outcomes and effect measures | 13 | State all treatment comparisons of interests. State all outcomes addressed and define them in detail. State whether they were pre-specified for the review and, if applicable, whether they were primary/main or secondary/additional outcomes. Give the principal measures of effect (such as risk ratio, hazard ratio, difference in means) used for each outcome. |  |
| Synthesis methods | 14 | Describe the meta-analysis methods used to synthesise IPD. Specify any statistical methods and models used. Issues should include (but are not restricted to):   - Use of a one-stage or two-stage approach. - How effect estimates were generated separately within each study and combined across studies (where applicable). - Specification of one-stage models (where applicable) including how clustering of patients within studies was accounted for. - Use of fixed or random effects models and any other model assumptions, such as proportional hazards. - How (summary) survival curves were generated (where applicable). - Methods for quantifying statistical heterogeneity (such as I^2^ and τ^2^). - How studies providing IPD and not providing IPD were analysed together (where applicable). - How missing data within the IPD were dealt with (where applicable). |  |
| Exploration of variation in effects | A2 | If applicable, describe any methods used to explore variation in effects by study or participant level characteristics (such as estimation of interactions between effect and covariates). State all participant-level characteristics that were analysed as potential effect modifiers, and whether these were pre-specified. |  |
| Risk of bias across studies | 15 | Specify any assessment of risk of bias relating to the accumulated body of evidence, including any pertaining to not obtaining IPD for particular studies, outcomes or other variables. |  |
| Additional analyses | 16 | Describe methods of any additional analyses, including sensitivity analyses. State which of these were pre-specified. |  |
| **Results** | | | |
| Study selection and IPD obtained | 17 | Give numbers of studies screened, assessed for eligibility, and included in the systematic review with reasons for exclusions at each stage. Indicate the number of studies and participants for which IPD were sought and for which IPD were obtained. For those studies where IPD were not available, give the numbers of studies and participants for which aggregate data were available. Report reasons for non-availability of IPD. Include a flow diagram. |  |
| Study characteristics | 18 | For each study, present information on key study and participant characteristics (such as description of interventions, numbers of participants, demographic data, unavailability of outcomes, funding source, and if applicable duration of follow-up). Provide (main) citations for each study. Where applicable, also report similar study characteristics for any studies not providing IPD. |  |
| IPD integrity | A3 | Report any important issues identified in checking IPD or state that there were none. |  |
| Risk of bias within studies | 19 | Present data on risk of bias assessments. If applicable, describe whether data checking led to the up-weighting or down-weighting of these assessments. Consider how any potential bias impacts on the robustness of meta-analysis conclusions. |  |
| Results of individual studies | 20 | For each comparison and for each main outcome (benefit or harm), for each individual study report the number of eligible participants for which data were obtained and show simple summary data for each intervention group (including, where applicable, the number of events), effect estimates and confidence intervals. These may be tabulated or included on a forest plot. |  |
| Results of syntheses | 21 | Present summary effects for each meta-analysis undertaken, including confidence intervals and measures of statistical heterogeneity. State whether the analysis was pre-specified, and report the numbers of studies and participants and, where applicable, the number of events on which it is based. |  |
|  |  | When exploring variation in effects due to patient or study characteristics, present summary interaction estimates for each characteristic examined, including confidence intervals and measures of statistical heterogeneity. State whether the analysis was pre-specified. State whether any interaction is consistent across trials. |  |
|  |  | Provide a description of the direction and size of effect in terms meaningful to those who would put findings into practice. |  |
| Risk of bias across studies | 22 | Present results of any assessment of risk of bias relating to the accumulated body of evidence, including any pertaining to the availability and representativeness of available studies, outcomes or other variables. |  |
| Additional analyses | 23 | Give results of any additional analyses (e.g. sensitivity analyses). If applicable, this should also include any analyses that incorporate aggregate data for studies that do not have IPD. If applicable, summarise the main meta-analysis results following the inclusion or exclusion of studies for which IPD were not available. |  |
| **Discussion** | | | |
| Summary of evidence | 24 | Summarise the main findings, including the strength of evidence for each main outcome. |  |
| Strengths and limitations | 25 | Discuss any important strengths and limitations of the evidence including the benefits of access to IPD and any limitations arising from IPD that were not available. |  |
| Conclusions | 26 | Provide a general interpretation of the findings in the context of other evidence. |  |
| Implications | A4 | Consider relevance to key groups (such as policy makers, service providers and service users). Consider implications for future research. |  |
| **Funding** | | | |
| Funding | 27 | Describe sources of funding and other support (such as supply of IPD), and the role in the systematic review of those providing such support. |  |

**A1 – A3 denote new items that are additional to standard PRISMA items. A4 has been created as a result of re-arranging content of the standard PRISMA statement to suit the way that systematic review IPD meta-analyses are reported.**

© Reproduced with permission of the PRISMA IPD Group, which encourages sharing and reuse for non-commercial purposes

# The Preferred Reporting Items for Systematic reviews and Meta-Analyses (PRISMA) statement – Diagnostic Test Accuracy Extension

| **Section/topic** | **#** | **PRISMA-DTA Checklist Item** | **Reported on page #** |
| --- | --- | --- | --- |
| **TITLE / ABSTRACT** | | |  |
| Title | 1 | Identify the report as a systematic review (+/- meta-analysis) of diagnostic test accuracy (DTA) studies. |  |
| Abstract | 2 | Abstract: See PRISMA-DTA for abstracts. |  |
| **INTRODUCTION** | | |  |
| Rationale | 3 | Describe the rationale for the review in the context of what is already known. |  |
| Clinical role of index test | D1 | State the scientific and clinical background, including the intended use and clinical role of the index test, and if applicable, the rationale for minimally acceptable test accuracy (or minimum difference in accuracy for comparative design). |  |
| Objectives | 4 | Provide an explicit statement of question(s) being addressed in terms of participants, index test(s), and target condition(s). |  |
| **METHODS** | | |  |
| Protocol and registration | 5 | Indicate if a review protocol exists, if and where it can be accessed (e.g., Web address), and, if available, provide registration information including registration number. |  |
| Eligibility criteria | 6 | Specify study characteristics (participants, setting, index test(s), reference standard(s), target condition(s), and study design) and report characteristics (e.g., years considered, language, publication status) used as criteria for eligibility, giving rationale. |  |
| Information sources | 7 | Describe all information sources (e.g., databases with dates of coverage, contact with study authors to identify additional studies) in the search and date last searched. |  |
| Search | 8 | Present full search strategies for all electronic databases and other sources searched, including any limits used, such that they could be repeated. |  |
| Study selection | 9 | State the process for selecting studies (i.e., screening, eligibility, included in systematic review, and, if applicable, included in the meta-analysis). |  |
| Data collection process | 10 | Describe method of data extraction from reports (e.g., piloted forms, independently, in duplicate) and any processes for obtaining and confirming data from investigators. |  |
| Definitions for data extraction | 11 | Provide definitions used in data extraction and classifications of target condition(s), index test(s), reference standard(s) and other characteristics (e.g. study design, clinical setting). |  |
| Risk of bias and applicability | 12 | Describe methods used for assessing risk of bias in individual studies and concerns regarding the applicability to the review question. |  |
| Diagnostic accuracy measures | 13 | State the principal diagnostic accuracy measure(s) reported (e.g. sensitivity, specificity) and state the unit of assessment (e.g. per-patient, per-lesion). |  |
| Synthesis of results | 14 | Describe methods of handling data, combining results of studies and describing variability between studies. This could include, but is not limited to: a) handling of multiple definitions of target condition. b) handling of multiple thresholds of test positivity, c) handling multiple index test readers, d) handling of indeterminate test results, e) grouping and comparing tests, f) handling of different reference standards |  |

Page 1 of 2

| **Section/topic** | **#** | **PRISMA-DTA Checklist Item** | **Reported on page #** |
| --- | --- | --- | --- |
| Meta-analysis | D2 | Report the statistical methods used for meta-analyses, if performed. |  |
| Additional analyses | 16 | Describe methods of additional analyses (e.g., sensitivity or subgroup analyses, meta-regression), if done, indicating which were pre-specified. |  |
| **RESULTS** | | |  |
| Study selection | 17 | Provide numbers of studies screened, assessed for eligibility, included in the review (and included in meta-analysis, if applicable) with reasons for exclusions at each stage, ideally with a flow diagram. |  |
| Study characteristics | 18 | For each included study provide citations and present key characteristics including: a) participant characteristics (presentation, prior testing), b) clinical setting, c) study design, d) target condition definition, e) index test, f) reference standard, g) sample size, h) funding sources |  |
| Risk of bias and applicability | 19 | Present evaluation of risk of bias and concerns regarding applicability for each study. |  |
| Results of individual studies | 20 | For each analysis in each study (e.g. unique combination of index test, reference standard, and positivity threshold) report 2x2 data (TP, FP, FN, TN) with estimates of diagnostic accuracy and confidence intervals, ideally with a forest or receiver operator characteristic (ROC) plot. |  |
| Synthesis of results | 21 | Describe test accuracy, including variability; if meta-analysis was done, include results and confidence intervals. |  |
| Additional analysis | 23 | Give results of additional analyses, if done (e.g., sensitivity or subgroup analyses, meta-regression; analysis of index test: failure rates, proportion of inconclusive results, adverse events). |  |
| **DISCUSSION** | | |  |
| Summary of evidence | 24 | Summarize the main findings including the strength of evidence. |  |
| Limitations | 25 | Discuss limitations from included studies (e.g. risk of bias and concerns regarding applicability) and from the review process (e.g. incomplete retrieval of identified research). |  |
| Conclusions | 26 | Provide a general interpretation of the results in the context of other evidence. Discuss implications for future research and clinical practice (e.g. the intended use and clinical role of the index test). |  |
| **FUNDING** | | |  |
| Funding | 27 | For the systematic review, describe the sources of funding and other support and the role of the funders. |  |

*Adapted From:*  McInnes MDF, Moher D, Thombs BD, McGrath TA, Bossuyt PM, The PRISMA-DTA Group (2018). Preferred Reporting Items for a Systematic Review and Meta-analysis of Diagnostic Test Accuracy Studies: The PRISMA-DTA Statement. JAMA. 2018 Jan 23;319(4):388-396. doi: 10.1001/jama.2017.19163.

For more information, visit: **www.prisma-statement.org**.

Page 2 of 2

# Search Strategy

Ovid (EMBASE and Medline)

1 exp Biopsy/

2 biopsy.mp. or biopsies.ti,ab.

3 biopsy.af.

4 1 or 2 or 3

5 MRI-TB.ti,ab.

6 MRI.ti,ab.

7 MRI*.ti,ab.

8 exp Magnetic Resonance Imaging/

9 magnetic resonance imag*.ti,ab.

10 magnetic resonance imaging.af.

11 or/5-10

12 prostate.ti,ab.

13 ((prostat*) adj2 (neoplasm* or cancer* or carcinoma* or tumor* or tumour*)).ti,ab.

14 exp Prostatic Neoplasms/

15 prostate.af.

16 or/12-15

17 4 and 11 and 16

Web of Science

TS=((( biops*)) AND (("magnetic resonance imaging" or MRI)) AND ((prostat*)) AND ((detection or diag*)))

Cochrane Library

(biops*):ab,ti and ('magnetic resonance' or mri) and (prostat*):ab,ti

Clinicaltrials.gov

Search terms included: “Prostate neoplasm” and other terms included “MRI, biopsy”

# Anticipated Codebook

| **Variable** | **Description** | **Values/ labels** | **NOTES** |
| --- | --- | --- | --- |
| **Study Characteristics** | | | |
| TrialID | Which trial did this record originate from? | 1= PRECISION  2= PRECISE | Study-level data |
| TrialName | Which trial did this record originate from? | String/ Free text | Study-level data |
| PatientID | Patient identifier, uniquely identifies individual in a trial. | Continuous Identifier | Patient-level data |
| PatientIPDID | Patient identifier for IPD | Continuous Identifier | Patient-level data |
| Study_Design | What is the study design of the trial? | String/ Free text | Study-level data |
| Study_nsite | Number of sites in study | Continuous  Min: 0  Missing: . | Study-level data |
| Study_site | Which site did the patient originate from? | String/ Free text | Patient-level data |
| Study_site_id | Site number of site patient originates from? | Continuous identifier | Patient-level data |
| Inclusion_criteria | Inclusion criteria of the study patient originates from. | String/ Free text | Study-level data |
| Exclusion_criteria | Exclusion criteria of the study patient originates from. | String/ Free text | Study-level data |
| Primary_outcome | Primary outcome of the study patient originates from. | String/ Free text | Study-level data |
| Secondary_outcome | Secondary outcome(s) of the study patient originates from. | String/ Free text | Study-level data |
| Recruitment_preriod | Recruitment period of the study patient originates from. | String/ Free text | Study-level data |
| Followup_Dura | Duration of follow-up (months) | Continuous  Min: 0  Missing: . | Patient-level data  Study-level data |
| Followup_protocol | Follow-up protocol of the study patient originates from. | String/ Free text | Study-level data |
| TRUS_operators | Number of TRUS biopsies operators in study patient originates from | Integer  Min: 1  Missing: . | Study-level data |
| TRUS_exp | Median number of TRUS biopsies performed per year by TRUS biopsy operators in study patient originates from | Continuous  Min: 1  Missing: . | Study-level data |
| MRI_biop_operators | Number of MRI targeted biopsy operators in study patient originates from | Integer  Min: 1  Missing: . | Study-level data |
| MRI_biop_exp | Median number of MRI targeted biopsies performed per year by MRI targeted biopsy operators in study patient originates from | Continuous  Min: 1  Missing: . | Study-level data |
| MRI_reporters | Number of radiologists reporting MRI in study patient originates from | Integer  Min: 1  Missing: . | Study-level data |
| MRI_report_exp | Median number of prostate MRIs reported per year by radiologists reporting MRI in study patient originates from | Continuous  Min: 1  Missing: . | Study-level data |
| Path | Number of pathologists reporting prostate specimens in study patient originates from | Integer  Min: 1  Missing: . | Study-level data |
| Path_exp | Median Number of patient’s prostate specimens analysed per year by pathologists reporting prostate specimens in study patient originates from | Continuous  Min: 1  Missing: . | Study-level data |
| **Patient characteristics** | | | |
| Assign_Intervention | Which intervention was the patient assigned to? | 0 = TRUS pathway  1 = MRI pathway | Patient-level data |
| Comp_Intervention | Which intervention did the patient undergo? | 0 = TRUS pathway  1 = MRI pathway | Patient-level data |
| Complete_followup | Did the patient complete follow-up? | 0 = Yes  1 = No | Patient-level data |
| Age | Age of participants in years | Continuous  Min: 0  Missing: . | Patient-level data |
| PSA_levels | PSA level of patient at enrolment in ng/ml | Continuous  Min: 0  Missing: . | Patient-level data |
| Prostate_Volume | Volume of prostate measured in mls (or cc?) at enrolment | Continuous  Min: 0  Missing: . | Patient-level data |
| PSA_Density | PSA density of patient at enrolment | Continuous  Min: 0  Missing: . | Patient-level data |
| Fam_hx | Any family history of PCa? | 0 = No  1 = Yes  2 = Do not know  . = Missing | Patient-level data |
| Abnormal_DRE | Abnormal DRE at point of enrolment? | 0 = No  1 = Yes  . = Missing | Patient-level data |
| PCa_Risk | Risk of Prostate Cancer according to Prostate Cancer Prevention Trial Risk Calculator, v2.0 | Continuous percentage (0-1.0)  Min: 0  Missing: . | Patient-level data |
| ECOG | ECOG Scale of Performance Status at the point of enrolment | 0 = Fully active, able to carry on all pre-disease performance without restriction  1 = Restricted in physically strenuous activity but ambulatory and able to carry out work of a light or sedentary nature, e.g., light house work, office work  2 = Ambulatory and capable of all selfcare but unable to carry out any work activities; up and about more than 50% of waking hours  3 = Capable of only limited selfcare; confined to bed or chair more than 50% of waking hours  4 = Completely disabled; cannot carry on any selfcare; totally confined to bed or chair  5 = Dead  . = Missing | Patient-level data |
| **MRI Arm** | | | |
| Field_strength | What is the field strength of MRI? | 1 = 1.5T  2= 3.0T  . = Missing | Patient-level data |
| MRI_sequence | What sequence was used? | 1 = Biparametric MRI  2 = Multiparametric MRI  . = Missing | Patient-level data |
| MRI_Biop_approach | What is the approach of MRI targeted biopsy | 1 = Tranperineal  2 = Transrectal  . = Missing | Patient-level data |
| MRI_Biop_Reg | What type of registration was used for the patient during MRI targeted biopsy? | 1 = Visual only  2 = Software-assisted only  3 = Both visual and software-assisted  . = Missing | Patient-level data |
| MRI_sus_score | What is the MRI suspicion score after MRI? | Likert Scale (1-5) | Patient-level data |
| PIRADS2_score | What is the PI-RADS v2 score? | 1 = very low (clinically significant cancer is highly unlikely to be present)  2 = low (clinically significant cancer is unlikely to be present)  3 = intermediate (the presence of clinically significant cancer is equivocal)  4 = high (clinically significant cancer is likely to be present)  5 = very high (clinically significant cancer is highly likely to be present)  X = component of exam technically inadequate or not performed  . = Missing | Patient-level data |
| Num_sus_lesions | How many suspicious lesions were found during MRI? | Integer  Min: 0  Missing: . | Patient-level data |
| Max_sus_score | Highest MRI score for men with suspicious lesion | 1 = 3  2 = 4  3 = 5  . = Missing | Patient-level data |
| MRI_lesion_diameter | Lesion diameter under MRI in mm | Continuous  Min: 0  Missing : . | Patient-level data |
| MRI_lesion_volume | Lesion volume under MRI in cc | Continuous  Min: 0  Missing : . | Patient-level data |
| **TRUS Arm** | | | |
| TRUS_lesion_volume | Lesion volume under TRUS in cc | Continuous  Min: 0  Missing :. | Patient-level data |
| TRUS_num_Biop | Number of biopsies taken under TRUS | Integer  Min: 0  Missing : . | Patient-level data |
| TRUS_time | Length of TRUS biopsy in mins | Continuous  Min: 0  Missing : . | Patient-level data |
| TRUS_ Anae | Anaesthetics used during TRUS | 1 = Local  2 = Sedation  3 = General  . = missing | Patient-level data |
| Outcomes | | | |
| Outcome_biopsy | What is the outcome of biopsy? | 1 = Biopsy excluded by MRI  2 = Benign  3 = Atypical small acinar proliferation  4 = High-grade prostatic intraepithelial neoplasia  . = Missing | Patient-level data |
| Gleason Score or Grade Group | What is the gleason score or GG for the patient? | 1 = 3+3/ GG1  2 = 3+4/ GG2  3 = 4+3/ GG3  4 = 3+5/ GG4  5 = 4+5/ 5+4/ 5+5/ GG5  6 = No biopsy  7 = Withdrawal  . = Missing | Patient-level data |
| CsPCa | Clinically Significant Prostate Cancer | 0 = No  1 = Yes | Patient-level data |
| CisPCa | Clinically Insignificant Prostate Cancer | 0 = No  1 = Yes | Patient-level data |
| Max Core Length | Maximum core length during biopsy? | Continuous  Min: 0  Missing: . | Patient-level data |
| Cores positive for cancer | Number of biopsy cores positive for cancer | Integer  Min: 0  Missing: . | Patient-level data |
| EQ5D_Baseline | Baseline EQ-5D score | Continuous  Min: 0  Missing: . | Patient-level data |
| EQ5D_FU | Post-treatment EQ-5D score | Continuous  Min: 0  Missing: . | Patient-level data |
| Pain_Score | Pain score post biopsy | Integer  Min:0  Max: 10  Missing: . | Patient-level data |
| Discomfort_Score | Discomfort Score after biopsy | Integer  MinL 0  Max: 10  Missing: . | Patient-level data |
| Post_op_comp_1_0 | Did the patient suffer any post-intervention complication within 30 days of intervention? | 0 = No  1 = Yes  . = Missing | Patient-level data |
| Post_op_comp | Number of post-intervention complications the patient suffered from within 30 days of intervention | Integer  Min: 0  . = Missing | Patient-level data |
| Post_op_comp_1 | 30-days post intervention complication - Fever | 0 = No  1= Yes  . = Missing | Patient-level data  N.B There may be complications or adverse events that may not be listed here and will need updating |
| Post_op_comp_2 | 30-days post intervention complication – Blood in urine | 0 = No  1= Yes  . = Missing | Patient-level data  N.B There may be complications or adverse events that may not be listed here and will need updating |
| Post_op_comp_3 | 30-days post intervention complication – Blood in semen | 0 = No  1= Yes  . = Missing | Patient-level data  N.B There may be complications or adverse events that may not be listed here and will need updating |
| Post_op_comp_4 | 30-days post intervention complication – Blood in the stools or from back passage | 0 = No  1= Yes  . = Missing | Patient-level data  N.B There may be complications or adverse events that may not be listed here and will need updating |
| Post_op_comp_5 | 30-days post intervention complication – Acute urinary retention | 0 = No  1= Yes  . = Missing | Patient-level data  N.B There may be complications or adverse events that may not be listed here and will need updating |
| Post_op_comp_6 | 30-days post intervention complication – Erectile dysfunction | 0 = No  1= Yes  . = Missing | Patient-level data  N.B There may be complications or adverse events that may not be listed here and will need updating |
| Post_op_comp_7 | 30-days post intervention complication – Urinary Tract infection | 0 = No  1= Yes  . = Missing | Patient-level data  N.B There may be complications or adverse events that may not be listed here and will need updating |
| Post_op_comp_8 | 30-days post intervention complication – Urinary retention | 0 = No  1= Yes  . = Missing | Patient-level data  N.B There may be complications or adverse events that may not be listed here and will need updating |
| Post_op_comp_9 | 30-days post intervention complication – Pain at site of procedure | 0 = No  1= Yes  . = Missing | Patient-level data  N.B There may be complications or adverse events that may not be listed here and will need updating |
| Post_op_comp_10 | 30-days post intervention complication – Men for whom another procedure will be a major problem | 0 = No  1= Yes  . = Missing | Patient-level data  N.B There may be complications or adverse events that may not be listed here and will need updating |
| AE_1_0 | Did the patient suffer any investigator-reported adverse events? | 0 = No  1= Yes  . = Missing | Patient-level data  N.B There may be complications or adverse events that may not be listed here and will need updating |
| AE_count | Number of investigator-reported adverse events has the patient suffered? | Integer  Min: 0  Missing: . | Patient-level data  N.B There may be complications or adverse events that may not be listed here and will need updating |
| AE_serious | Did the patient suffer from any serious investigator-reported adverse events? | 0 = No  1= Yes  . = Missing | Patient-level data  N.B There may be complications or adverse events that may not be listed here and will need updating |
| AE_serious_count | Number of serious investigator-reported adverse events has the patient suffered | Integer  Min: 0  Missing: . | Patient-level data  N.B There may be complications or adverse events that may not be listed here and will need updating |
| AE_1 | Investigator reported adverse events – Sepsis | 0 = No  1= Yes  . = Missing | Patient-level data  N.B There may be complications or adverse events that may not be listed here and will need updating |
| AE_2 | Investigator reported adverse events – Haematuria | 0 = No  1= Yes  . = Missing | Patient-level data  N.B There may be complications or adverse events that may not be listed here and will need updating |
| AE_3 | Investigator reported adverse events – Prostatitis | 0 = No  1= Yes  . = Missing | Patient-level data  N.B There may be complications or adverse events that may not be listed here and will need updating |
| AE_4 | Investigator reported adverse events (unrelated to intervention) – Fatigue | 0 = No  1= Yes  . = Missing | Patient-level data  N.B There may be complications or adverse events that may not be listed here and will need updating |
| AE_5 | Investigator reported adverse events (unrelated to intervention) – Runny nose and cough | 0 = No  1= Yes  . = Missing | Patient-level data  N.B There may be complications or adverse events that may not be listed here and will need updating |
| AE_6 | Investigator reported adverse events (unrelated to intervention) – Myocardial Infarction | 0 = No  1= Yes  . = Missing | Patient-level data  N.B There may be complications or adverse events that may not be listed here and will need updating |
| AE_7 | Investigator reported adverse events (unrelated to intervention) – Pulmonary Embolism | 0 = No  1= Yes  . = Missing | Patient-level data  N.B There may be complications or adverse events that may not be listed here and will need updating |
| AE_8 | Investigator reported adverse events (unrelated to intervention) – Death | 0 = No  1= Yes  . = Missing | Patient-level data  N.B There may be complications or adverse events that may not be listed here and will need updating |
| Treatment | Definitive treatment undergone by patient | 1 = Discharge patient, no treatment or follow-up required  2 = PSA monitoring  3 = Active Surveillance  (Radical treatments)  4 = Radical Prostatectomy  5 = Radiotherapy +/- neoadjuvant hormone therapy  6 = Brachy therapy  7 = Focal therapy  8 = Hormone therapy +/- chemotherapy  9 = Watchful waiting  10 = Other  11 = Unknown  . = Missing | Patient-level data |
| Further_Diag | Further diagnostic tests ordered from treatment decision visit | 0 = None  1 = mpMRI – patient choice  2 = mpMRI – Clinician choice  3 = Immediate further prostate biopsies – patient choice  4 = Immediate further prostate biopsies – clinician choice  5 = MRI for staging  6 = Bone scan  7 = CT  8= PET-CT  9= PET-MRI  . = Missing | Patient-level data |

**QUADAS-C tailored assessment and RoB 2.0 for assessing bias in VISION study: An indiVidual patient data meta-analysis of randomised trials comparing MRI-targeted biopsy to standard transrectal ultraSound guided bIopsy in the detection of prOstate caNcer**

**Version 1.0 13/04/2021**

**Review Question: from protocol V 1.2**

For the purposes of the QUADAS-C assessment, the main outcome of the study should be taken in consideration, rather than any subgroup analyses.

Patients:

- Men with clinical suspicion or confirmed prostate cancer (e.g. raised PSA, abnormal DRE, family history of prostate cancer) undergoing further diagnostic biopsies of the prostate for prostate cancer
- Biopsy-naïve men only

Index Test 1:

- MRI-targeted biopsy (done by any registration technique)

Index Test 2 (Comparator):

- Standard 10-12 core trans-rectal or trans-perineal ultrasound guided systematic biopsies

Reference standard:

- Not applicable

Acknowledgement about limitations of diagnostic accuracy evaluation in prostate cancer

- Prostate cancer is a difficult disease for diagnostic accuracy evaluation as there is no adequate reference standard that can be carried out in all men.
- Radical prostatectomy is only carried out in a small proportion of men diagnosed with high risk disease on initial biopsy so is an imperfect reference standard.
- It is accepted that standard TRUS biopsy and transperineal template biopsy have limitations as reference tests so a comparison of cancer detected between MRI-targeted biopsy and the systematic biopsy will form the basis for test evaluation.
- Where transperineal template biopsy has been carried out with high sampling density e.g. 5mm or high sampling density it may be used as an acceptable reference standard to evaluate diagnostic accuracy

Reference standard and target condition

- Primary target condition: Presence or absence of clinically significant prostate cancer
- Reference standard: Not Applicable

**Example flow diagrams for typical studies to be included in this review**


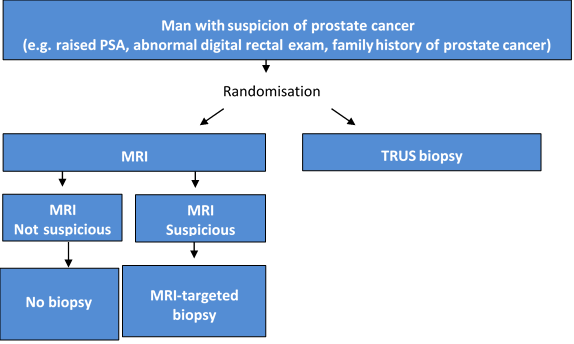


Note: not all randomised studies will follow the same format. For example men with negative MRI may undergo no biopsy or men with positive MRI may only undergo targeted biopsy. These studies are excluded from this review.

Note also: multiple comparisons between MRI-targeted and systematic biopsy in these types of study designs may be possible

# QUADAS-C Tool

| **Domain 1: Patient selection** | | |
| --- | --- | --- |
| RISK OF BIAS: Could selection of patients have introduced bias? | | |
| Describe the methods of patient selection briefly: | | |
| ***Single test accuracy (QUADAS-2)*** | ***Answers for MRI arm*** | ***Answers for TRUS arm*** |
| **Signalling Questions** | | |
| 1.1: Was a consecutive or random sample of patients enrolled? | Yes / No / Unclear | Yes / No / Unclear |
| 1.2: Did the study avoid inappropriate exclusions?  Inappropriate exclusions would be exclusion of patients who are more or less likely to have disease which may influence the diagnostic yield of the test.  Examples of inappropriate exclusions:   - excluding patients with likely T3/T4 or extremely high PSA would be inappropriate - including only patients who underwent radical prostatectomy   Studies that avoid inappropriate exclusions are studies that have been as broad as possible in terms of the population included. | Yes / No / Unclear | Yes / No / Unclear |
| **Risk of Bias** | | |
| 1.3: Could the selection of patients have introduced bias?  High risk if ‘No’ for at least one SQ  Low risk if ‘Yes’ for all SQs.  Unclear if “Unclear” for at least one SQ (though “No” for one SQ supersedes “Unclear” if both results present). | Low risk / High Risk / Unclear | Low risk / High Risk / Unclear |
| Concerns regarding applicability | | |
| Describe briefly the included patients (prior testing, presentation, intended use of index test and setting): | | |
| Are there concerns that the included patients and setting do not match the review question?  Are the population included representative of patients in clinical practice? (i.e PSA level, stage and baseline characteristics) | Low risk / High Risk / Unclear | Low risk / High Risk / Unclear |
| ***Comparative Accuracy*** | ***Answers for the comparison of MRI arm and TRUS arm*** | |
| **Signalling questions** | | |
| C1.1: Was risk of bias for this domain judged “low” for both index tests? | Yes/No/Unclear | |
| C1.2: Was the allocation sequence random? | Yes/No/Unclear | |
| C1.3: Was the allocation sequence concealed until patients were enrolled and assigned to index tests? | Yes/No/Unclear | |
| **Signalling questions** | | |
| C1.4: Could the selection of patients have introduced bias in the comparison?  High risk if ‘No’ for at least one SQ  Low risk if ‘Yes’ for all SQs.  Unclear if “Unclear” for at least one SQ (though “No” for one SQ supersedes “Unclear” if both results present). | Low/ High/ Unclear | |

| **Domain 2: Index Test** | | |
| --- | --- | --- |
| RISK OF BIAS: Could the conduct or interpretation of the index test have introduced bias? | | |
| Describe briefly the nature of the MRI-targeted biopsy, how it was conducted and results interpreted: | | |
| ***Single test accuracy (QUADAS-2)*** | ***Answers for MRI arm*** | ***Answers for TRUS arm*** |
| ***Signalling Questions*** | | |
| 2.1: Was the MRI-targeted biopsy performed without knowledge of the results of the systematic biopsy?  In a RCT setting of this review, this is unlikely, hence most of the time this will be “Yes”. | Yes / No / Unclear | Yes / No / Unclear |
| 2.2: Was the MRI score / risk threshold for patients to undergo targeted biopsy pre-specified? | Yes / No / Unclear | Yes / No / Unclear |
| **Risk of bias** |  |  |
| 2.3: Could the conduct or interpretation of the index test have introduced bias?  High risk if ‘No’ for at least one SQ  Low risk if ‘Yes’ for all SQs.  Unclear if “Unclear” for at least one SQ (though “No” for one SQ supersedes “Unclear” if both results present). | Low risk / High Risk / Unclear | Low risk / High Risk / Unclear |
| **Concerns regarding applicability** | | |
| Are there concerns that the index test, its conduct, or interpretation differ from the review question?  Are the index tests included in the study representative of those in clinical practice? (i.e expertise and access to equipment) | Low risk / High Risk / Unclear | Low risk / High Risk / Unclear |
| **Comparative accuracy (QUADAS-C)** | **Answers for the comparison of MRI arm vs TRUS arm** | |
| **Signalling questions** | | |
| C2.1: Was risk of bias for this domain judged ‘low’ for all index tests? | Yes/No/Unclear | |
| C2.2: Were differences in the conduct or interpretation between the index tests unlikely to advantage one of the tests? | Yes/No/Unclear | |
| **Risk of bias** | | |
| C2.3: Could the conduct or interpretation of the index tests have introduced bias in the comparison?  High risk if ‘No’ for at least one applicable SQ  Low risk if ‘Yes’ for all applicable SQs.  Unclear if “Unclear” for at least one applicable SQ. (Though “No” for one SQ supersedes “Unclear” if both results present). | Low risk / High Risk / Unclear | |

Reference Standard domain was deemed irrelevant to the study as there are no reference tests, thus has been excluded from this review.

| **Domain 3: Flow and Timing** | | |
| --- | --- | --- |
| RISK OF BIAS: - Could the patient flow have introduced bias? | | |
| Describe briefly the nature of the interventions, how it was conducted and results interpreted: | | |
| ***Single test accuracy (QUADAS-2)*** | ***Answers for MRI arm*** | ***Answers for TRUS arm*** |
| **Signalling Questions** | | |
| 3.1: Was the time interval between mpMRI and MRI targeted biopsy less than 6 months? | Yes / No / Unclear | Yes / No / Unclear |
| 3.2: Were all patients who underwent testing included in the analysis?  Please look out for withdrawal numbers and lost to follow-up patients within each study arm: is the number of patients significantly different between arms? If there are imbalances between arms, please answer “No” | Yes / No / Unclear | Yes / No / Unclear |
| Risk of bias |  | |
| 3.3: Could the patient flow have introduced bias?  High risk if ‘No’ for at least one SQ  Low risk if ‘Yes’ for all SQs.  Unclear if “Unclear” for at least one SQ. (Though “No” for one SQ supersedes “Unclear” if both results present). | Low risk / High Risk / Unclear | Low risk / High Risk / Unclear |
| **Comparative accuracy (QUADAS-C)** | **Answers for the comparison of MRI arm vs TRUS arm** | |
| **Signalling questions** | | |
| C3.1: Was risk of bias for this domain judged ‘low’ for all index tests? | Yes/No/Unclear | |
| C3.2: Are the proportions and reasons for missing data similar across index tests? | Yes/No/Unclear | |
| **Risk of bias** | | |
| C3.4: Could the patient flow have introduced bias in the comparison?  High risk if ‘No’ for at least one applicable SQ  Low risk if ‘Yes’ for all applicable SQs.  Unclear if “Unclear” for at least one applicable SQ. (Though “No” for one SQ supersedes “Unclear” if both results present). | Low/ High/ Unclear | |

# RoB 2.0 tool

taken from Sterne JAC, Savović J, Page MJ, Elbers RG, Blencowe NS, Boutron I, et al. RoB 2: a revised tool for assessing risk of bias in randomised trials. BMJ. 2019;366:l4898.

Revised Cochrane risk-of-bias tool for randomized trials (RoB 2)

SHORT VERSION (CRIBSHEET)

Edited by Julian PT Higgins, Jelena Savović, Matthew J Page, Jonathan AC Sterne on behalf of the RoB 2 Development Group

**Version of 22 August 2019**

The development of the RoB 2 tool was supported by the MRC Network of Hubs for Trials Methodology Research (MR/L004933/2- N61), with the support of the host MRC ConDuCT-II Hub (Collaboration and innovation for Difficult and Complex randomised controlled Trials In Invasive procedures - MR/K025643/1), by MRC research grant MR/M025209/1, and by a grant from The Cochrane Collaboration.


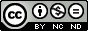


This work is licensed under a [Creative Commons Attribution-NonCommercial-NoDerivatives 4.0 International License.](http://creativecommons.org/licenses/by-nc-nd/4.0/)

**Preliminary considerations**

| **Study design**   - Individually-randomized parallel-group trial - Cluster-randomized parallel-group trial - Individually randomized cross-over (or other matched) trial      \|  \| Comparator: \|  \| \| --- \| --- \| --- \|   **For the purposes of this assessment, the interventions being compared are defined as** Experimental:     \|  \| \| --- \|   **Specify which outcome is being assessed for risk of bias**     \|  \| \| --- \|   **Specify the numerical result being assessed.** In case of multiple alternative analyses being presented, specify the numeric result (e.g. RR = 1.52 (95% CI 0.83 to 2.77) and/or a reference (e.g. to a table, figure or paragraph) that uniquely defines the result being assessed.    **Is the review team’s aim for this result…?**   - to assess the effect of *assignment to intervention* (the ‘intention-to-treat’ effect) - to assess the effect of *adhering to intervention* (the ‘per-protocol’ effect)     **If the aim is to assess the effect of *adhering to intervention***, select the deviations from intended intervention that should be addressed (at least one must be checked):   - occurrence of non-protocol interventions - failures in implementing the intervention that could have affected the outcome - non-adherence to their assigned intervention by trial participants |
| --- | --- | --- | --- | --- | --- |

| **Which of the following sources were obtained to help inform the risk-of-bias assessment? (tick as many as apply)**   - Journal article(s) - Trial protocol - Statistical analysis plan (SAP) - Non-commercial trial registry record (e.g. ClinicalTrials.gov record) - Company-owned trial registry record (e.g. GSK Clinical Study Register record) ⬜ “Grey literature” (e.g. unpublished thesis) - Conference abstract(s) about the trial - Regulatory document (e.g. Clinical Study Report, Drug Approval Package) - Research ethics application - Grant database summary (e.g. NIH RePORTER or Research Councils UK Gateway to Research) ⬜ Personal communication with trialist - Personal communication with the sponsor |
| --- |

Domain 1: Risk of bias arising from the randomization process

| **Signalling questions** | **Elaboration** | **Response options** |
| --- | --- | --- |
| **1.1 Was the allocation sequence random?** | Answer ‘Yes’ if a random component was used in the sequence generation process. Examples include computer-generated random numbers; reference to a random number table; coin tossing; shuffling cards or envelopes; throwing dice; or drawing lots. Minimization is generally implemented with a random element (at least when the scores are equal), so an allocation sequence that is generated using minimization should generally be considered to be random.  Answer ‘No’ if no random element was used in generating the allocation sequence or the sequence is predictable. Examples include alternation; methods based on dates (of birth or admission); patient record numbers; allocation decisions made by clinicians or participants; allocation based on the availability of the intervention; or any other systematic or haphazard method.  Answer ‘No information’ if the only information about randomization methods is a statement that the study is randomized.  In some situations a judgement may be made to answer ‘Probably no’ or ‘Probably yes’. For example, , in the context of a large trial run by an experienced clinical trials unit, absence of specific information about generation of the randomization sequence, in a paper published in a journal with rigorously enforced word count limits, is likely to result in a response of ‘Probably yes’ rather than ‘No information’. Alternatively, if other (contemporary) trials by the same investigator team have clearly used non-random sequences, it might be reasonable to assume that the current study was done using similar methods. | Y/PY/PN/N/NI |
| **1.2 Was the allocation sequence concealed until participants were enrolled and assigned to interventions?** | Answer ‘Yes’ if the trial used any form of remote or centrally administered method to allocate interventions to participants, where the process of allocation is controlled by an external unit or organization, independent of the enrolment personnel (e.g. independent central pharmacy, telephone or internet-based randomization service providers).  Answer ‘Yes’ if envelopes or drug containers were used appropriately. Envelopes should be opaque, sequentially numbered, sealed with a tamper-proof seal and opened only after the envelope has been irreversibly assigned to the participant. Drug containers should be sequentially numbered and of identical appearance, and dispensed or administered only after they have been irreversibly assigned to the participant. This level of detail is rarely provided in reports, and a judgement may be required to justify an answer of ‘Probably yes’ or ‘Probably no’.  Answer ‘No’ if there is reason to suspect that the enrolling investigator or the participant had knowledge of the forthcoming allocation. | Y/PY/PN/N/NI |

| **1.3 Did baseline differences between intervention groups suggest a problem with the randomization process?** | *Note that differences that are compatible with chance do not lead to a risk of bias. A small number of differences identified as ‘statistically significant’ at the conventional 0.05 threshold should usually be considered to be compatible with chance.*  Answer ‘No’ if no imbalances are apparent or if any observed imbalances are compatible with chance.  Answer ‘Yes’ if there are imbalances that indicate problems with the randomization process, including:   1. substantial differences between intervention group sizes, compared with the intended allocation ratio; or 2. a substantial excess in statistically significant differences in baseline characteristics between intervention groups, beyond that expected by chance; or 3. imbalance in one or more key prognostic factors, or baseline measures of outcome variables, that is very unlikely to be due to chance and for which the between-group difference is big enough to result in bias in the intervention effect estimate.   Also answer ‘Yes’ if there are other reasons to suspect that the randomization process was problematic:   1. excessive similarity in baseline characteristics that is not compatible with chance.   Answer ‘No information’ when there is no *useful* baseline information available (e.g. abstracts, or studies that reported only baseline characteristics of participants in the final analysis).  The answer to this question should not influence answers to questions 1.1 or 1.2. For example, if the trial has large baseline imbalances, but authors report adequate randomization methods, questions 1.1 and 1.2 should still be answered on the basis of the reported adequate methods, and any concerns about the imbalance should be raised in the answer to the question 1.3 and reflected in the domain-level risk-ofbias judgement.  Trialists may undertake analyses that attempt to deal with flawed randomization by controlling for imbalances in prognostic factors at baseline. To remove the risk of bias caused by problems in the randomization process, it would be necessary to know, and measure, all the prognostic factors that were imbalanced at baseline. It is unlikely that all important prognostic factors are known and measured, so such analyses will at best reduce the risk of bias. If review authors wish to assess the risk of bias in a trial that controlled for baseline imbalances in order to mitigate failures of randomization, the study should be assessed using the ROBINS-I tool. | Y/PY/PN/N/NI |
| --- | --- | --- |
| **Risk-of-bias judgement** | See algorithm. | Low / High / Some concerns |
| Optional: What is the predicted direction of bias arising from the randomization process? | If the likely direction of bias can be predicted, it is helpful to state this. The direction might be characterized either as being towards (or away from) the null, or as being in favour of one of the interventions. | NA / Favours experimental /  Favours comparator /  Towards null /Away from null /  Unpredictable |


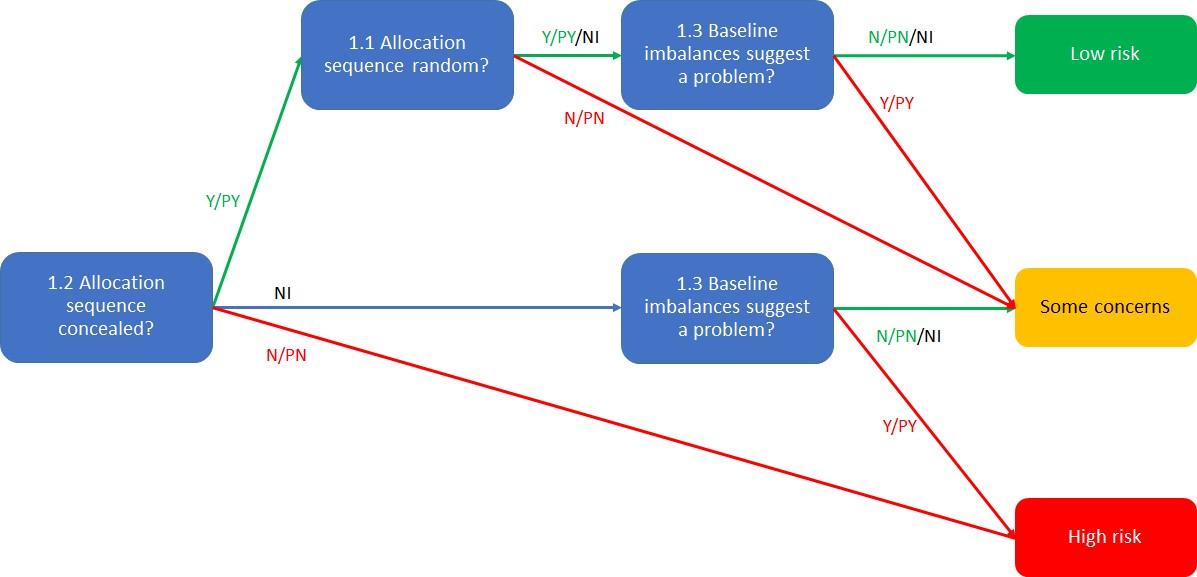


**Algorithm for suggested judgement of risk of bias arising from the randomization process**

Domain 2: Risk of bias due to deviations from the intended interventions (*effect of assignment to intervention*)

| **Signalling questions** | **Elaboration** | **Response options** |
| --- | --- | --- |
| **2.1. Were participants aware of their assigned intervention during the trial?** | If participants are aware of their assigned intervention it is more likely that health-related behaviours will differ between the intervention groups. Blinding participants, most commonly through use of a placebo or sham intervention, may prevent such differences. If participants experienced side effects or toxicities that they knew to be specific to one of the interventions, answer this question ‘Yes’ or ‘Probably yes’. | Y/PY/PN/N/NI |
| **2.2. Were carers and people delivering the interventions aware of participants' assigned intervention during the trial?** | If carers or people delivering the interventions are aware of the assigned intervention then its implementation, or administration of non-protocol interventions, may differ between the intervention groups. Blinding may prevent such differences. If participants experienced side effects or toxicities that carers or people delivering the interventions knew to be specific to one of the interventions, answer question ‘Yes’ or ‘Probably yes’. If randomized allocation was not concealed, then it is likely that carers and people delivering the interventions were aware of participants' assigned intervention during the trial. | Y/PY/PN/N/NI |

| **2.3. If Y/PY/NI to 2.1 or 2.2: Were there deviations from the intended intervention that arose because of the trial context?** | For the effect of assignment to intervention, this domain assesses problems that arise when changes from assigned intervention that are inconsistent with the trial protocol arose because of the trial context. We use the term **trial context** to refer to effects of recruitment and engagement activities on trial participants and when trial personnel (carers or people delivering the interventions) undermine the implementation of the trial protocol in ways that would not happen outside the trial. For example, the process of securing informed consent may lead participants subsequently assigned to the comparator group to feel unlucky and therefore seek the experimental intervention, or other interventions that improve their prognosis.  Answer ‘Yes’ or ‘Probably yes’ **only** if there is evidence, or strong reason to believe, that the trial context led to failure to implement the protocol interventions or to implementation of interventions not allowed by the protocol.  Answer ‘No’ or ‘Probably no’ if there were changes from assigned intervention that are inconsistent with the trial protocol, such as non-adherence to intervention, but these are consistent with what could occur outside the trial context.  Answer ‘No’ or ‘Probably no’ for changes to intervention that are consistent with the trial protocol, for example cessation of a drug intervention because of acute toxicity or use of additional interventions whose aim is to treat consequences of one of the intended interventions.  If blinding is compromised because participants report side effects or toxicities that are specific to one of the interventions, answer ‘Yes’ or ‘Probably yes’ only if there were changes from assigned intervention that are inconsistent with the trial protocol and arose because of the trial context.  The answer ‘No information’ may be appropriate, because trialists do not always report whether deviations arose because of the trial context. | NA/Y/PY/PN/N/NI |
| --- | --- | --- |
| **2.4 If Y/PY to 2.3: Were these deviations likely to have affected the outcome?** | Changes from assigned intervention that are inconsistent with the trial protocol and arose because of the trial context will impact on the intervention effect estimate if they affect the outcome, but not otherwise. | NA/Y/PY/PN/N/NI |
| **2.5. If Y/PY/NI to 2.4: Were these deviations from intended intervention balanced between groups?** | Changes from assigned intervention that are inconsistent with the trial protocol and arose because of the trial context are more likely to impact on the intervention effect estimate if they are not balanced between the intervention groups. | NA/Y/PY/PN/N/NI |
| **2.6 Was an appropriate analysis used to estimate the effect of assignment to intervention?** | Both intention-to-treat (ITT) analyses and modified intention-to-treat (mITT) analyses excluding participants with missing outcome data should be considered appropriate. Both naïve ‘per-protocol’ analyses (excluding trial participants who did not receive their assigned intervention) and ‘as treated’ analyses (in which trial participants are grouped according to the intervention that they received, rather than according to their assigned intervention) should be considered inappropriate. Analyses excluding eligible trial participants post-randomization should also be considered inappropriate, but postrandomization exclusions of ineligible participants (when eligibility was not confirmed until after randomization, and could not have been influenced by intervention group assignment) can be considered appropriate. | Y/PY/PN/N/NI |
| **2.7 If N/PN/NI to 2.6: Was there potential for a substantial impact (on the result) of the failure to analyse participants in the group to which they were randomized?** | This question addresses whether the number of participants who were analysed in the wrong intervention group, or excluded from the analysis, was sufficient that there could have been a substantial impact on the result. It is not possible to specify a precise rule: there may be potential for substantial impact even if fewer than 5% of participants were analysed in the wrong group or excluded, if the outcome is rare or if exclusions are strongly related to prognostic factors. | NA/Y/PY/PN/N/NI |
| **Risk-of-bias judgement** | See algorithm. | Low / High / Some concerns |
| Optional: What is the predicted direction of bias due to deviations from intended interventions? | If the likely direction of bias can be predicted, it is helpful to state this. The direction might be characterized either as being towards (or away from) the null, or as being in favour of one of the interventions. | NA / Favours experimental / Favours comparator / Towards  null /Away from null /  Unpredictable |


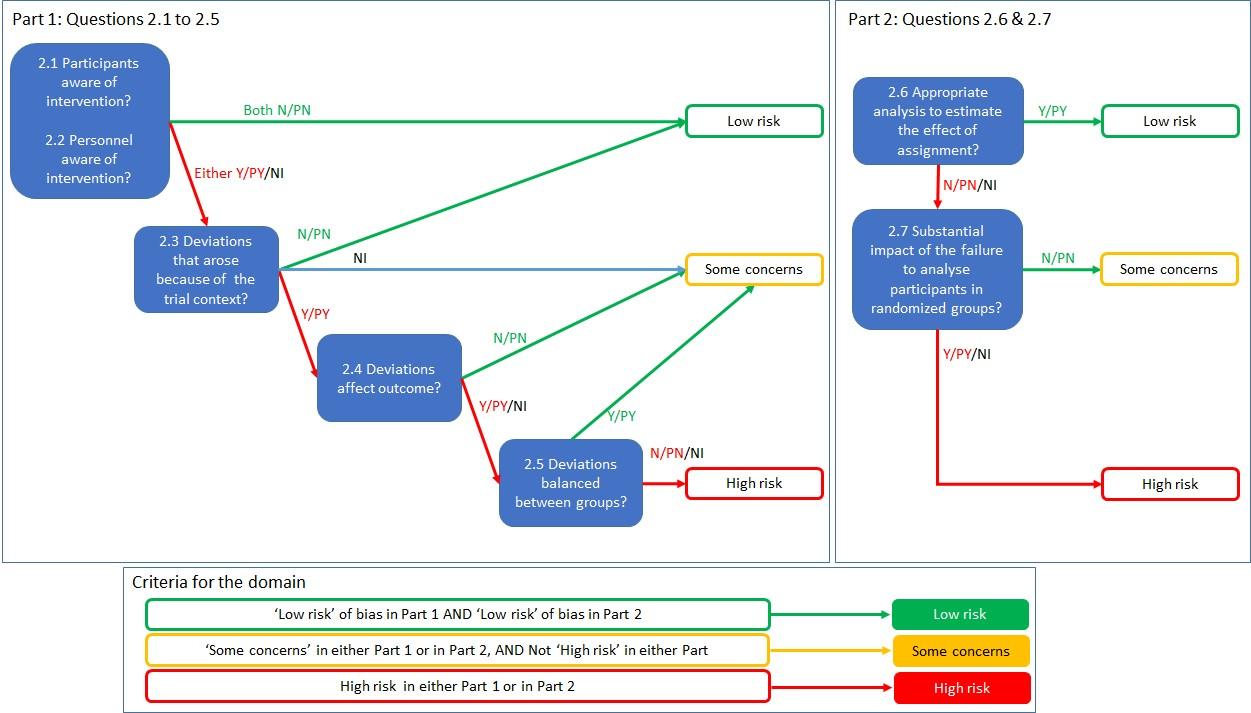


**Algorithm for suggested judgement of risk of bias due to deviations from the intended interventions (effect of assignment to intervention)**

Domain 2: Risk of bias due to deviations from the intended interventions (*effect of adhering to intervention*)

| **Signalling questions** | **Elaboration** | **Response options** |
| --- | --- | --- |
| **2.1. Were participants aware of their assigned intervention during the trial?** | If participants are aware of their assigned intervention it is more likely that health-related behaviours will differ between the intervention groups. Blinding participants, most commonly through use of a placebo or sham intervention, may prevent such differences. If participants experienced side effects or toxicities that they knew to be specific to one of the interventions, answer this question ‘Yes’ or ‘Probably yes’. | Y/PY/PN/N/NI |
| **2.2. Were carers and people delivering the interventions aware of participants' assigned intervention during the trial?** | If carers or people delivering the interventions are aware of the assigned intervention then its implementation, or administration of non-protocol interventions, may differ between the intervention groups. Blinding may prevent such differences. If participants experienced side effects or toxicities that carers or people delivering the interventions knew to be specific to one of the interventions, answer ‘Yes’ or ‘Probably yes’. If randomized allocation was not concealed, then it is likely that carers and people delivering the interventions were aware of participants' assigned intervention during the trial. | Y/PY/PN/N/NI |
| **2.3. [If applicable:] If Y/PY/NI to 2.1 or 2.2: Were important nonprotocol interventions balanced across intervention groups?** | This question is asked only if the preliminary considerations specify that the assessment will address imbalance of important non-protocol interventions between intervention groups. Important nonprotocol interventions are the additional interventions or exposures that: (1) are inconsistent with the trial protocol; (2) trial participants might receive with or after starting their assigned intervention; and (3) are prognostic for the outcome. Risk of bias will be higher if there is imbalance in such interventions between the intervention groups. | NA/Y/PY/PN/N/NI |
| **2.4. [If applicable:] Were there failures in implementing the intervention that could have affected the outcome?** | This question is asked only if the preliminary considerations specify that the assessment will address failures in implementing the intervention that could have affected the outcome. Risk of bias will be higher if the intervention was not implemented as intended by, for example, the health care professionals delivering care. Answer ‘No’ or ‘Probably no’ if implementation of the intervention was successful for most participants. | NA/Y/PY/PN/N/NI |
| **2.5. [If applicable:] Was there non-adherence to the assigned intervention regimen that could have affected participants’ outcomes?** | This question is asked only if the preliminary considerations specify that the assessment will address nonadherence that could have affected participants’ outcomes. Non-adherence includes imperfect compliance with a sustained intervention, cessation of intervention, crossovers to the comparator intervention and switches to another active intervention. Consider available information on the proportion of study participants who continued with their assigned intervention throughout follow up, and answer ‘Yes’ or ‘Probably yes’ if the proportion who did not adhere is high enough to raise concerns. Answer ‘No’ for studies of interventions that are administered once, so that imperfect adherence is not possible, and all or most participants received the assigned intervention. | NA/Y/PY/PN/N/NI |
| **2.6. If N/PN/NI to 2.3, or Y/PY/NI to 2.4 or 2.5: Was an appropriate analysis used to estimate the effect of adhering to the intervention?** | Both ‘ naïve ‘per-protocol’ analyses (excluding trial participants who did not receive their allocated intervention) and ‘as treated’ analyses (comparing trial participants according to the intervention they actually received) will usually be inappropriate for estimating the effect of adhering to intervention (the ‘per-protocol’ effect). However, it is possible to use data from a randomized trial to derive an unbiased estimate of the effect of adhering to intervention. Examples of appropriate methods include: (1) instrumental variable analyses to estimate the effect of receiving the assigned intervention in trials in which a single intervention, administered only at baseline and with all-or-nothing adherence, is compared with standard care; and (2) inverse probability weighting to adjust for censoring of participants who cease adherence to their assigned intervention, in trials of sustained treatment strategies. These methods depend on strong assumptions, which should be appropriate and justified if the answer to this question is ‘Yes’ or ‘Probably yes’. It is possible that a paper reports an analysis based on such methods without reporting information on the deviations from intended intervention, but it would be hard to judge such an analysis to be appropriate in the absence of such information.  If an important non-protocol intervention was administered to all participants in one intervention group, adjustments cannot be made to overcome this.  Some examples of analysis strategies that would not be appropriate to estimate the effect of adhering to intervention are (i) ‘Intention to treat (ITT) analysis’, (ii) ‘per protocol analysis’, (iii) ‘as-treated analysis’, (iv) ‘analysis by treatment received’. | NA/Y/PY/PN/N/NI |
| **Risk-of-bias judgement** | See algorithm. | Low / High / Some concerns |
| Optional: What is the predicted direction of bias due to deviations from intended interventions? | If the likely direction of bias can be predicted, it is helpful to state this. The direction might be characterized either as being towards (or away from) the null, or as being in favour of one of the interventions. | NA / Favours experimental / Favours comparator / Towards  null /Away from null /  Unpredictable |


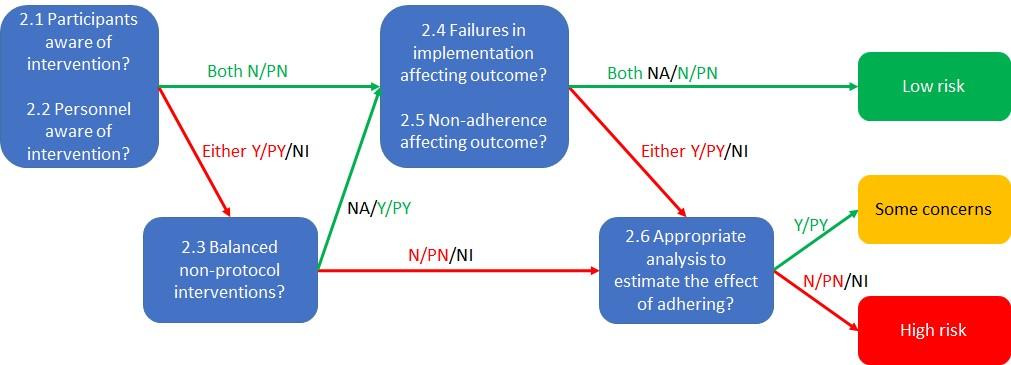


**Algorithm for suggested judgement of risk of bias due to deviations from the intended interventions (effect of adhering to intervention)**

## Domain 3: Risk of bias due to missing outcome data

| **Signalling questions** | **Elaboration** | **Response options** |
| --- | --- | --- |
| **3.1 Were data for this outcome available for all, or nearly all, participants randomized?** | The appropriate study population for an analysis of the intention to treat effect is all randomized participants.  “Nearly all” should be interpreted as that the number of participants with missing outcome data is sufficiently small that their outcomes, whatever they were, could have made no important difference to the estimated effect of intervention.  For continuous outcomes, availability of data from 95% of the participants will often be sufficient. For dichotomous outcomes, the proportion required is directly linked to the risk of the event. If the observed number of events is much greater than the number of participants with missing outcome data, the bias would necessarily be small.  Only answer ‘No information’ if the trial report provides no information about the extent of missing outcome data. This situation will usually lead to a judgement that there is a high risk of bias due to missing outcome data.  Note that imputed data should be regarded as missing data, and not considered as ‘outcome data’ in the context of this question. | Y/PY/PN/N/NI |
| **3.2 If N/PN/NI to 3.1: Is there evidence that the result was not biased by missing outcome data?** | Evidence that the result was not biased by missing outcome data may come from: (1) analysis methods that correct for bias; or (2) sensitivity analyses showing that results are little changed under a range of plausible assumptions about the relationship between missingness in the outcome and its true value. However, imputing the outcome variable, either through methods such as ‘last-observation-carriedforward’ or via multiple imputation based only on intervention group, should not be assumed to correct for bias due to missing outcome data. | NA/Y/PY/PN/N |
| **3.3 If N/PN to 3.2: Could missingness in the outcome depend on its true value?** | If loss to follow up, or withdrawal from the study, could be related to participants’ health status, then it is possible that missingness in the outcome was influenced by its true value. However, if all missing outcome data occurred for documented reasons that are unrelated to the outcome then the risk of bias due to missing outcome data will be low (for example, failure of a measuring device or interruptions to routine data collection).  In time-to-event analyses, participants censored during trial follow-up, for example because they withdrew from the study, should be regarded as having missing outcome data, even though some of their follow up is included in the analysis. Note that such participants may be shown as included in analyses in CONSORT flow diagrams. | NA/Y/PY/PN/N/NI |
| **3.4 If Y/PY/NI to 3.3: Is it likely that missingness in the outcome depended on its true value?** | This question distinguishes between situations in which (i) missingness in the outcome could depend on its true value (assessed as ‘Some concerns’) from those in which (ii) it is likely that missingness in the outcome depended on its true value (assessed as ‘High risk of bias’). Five reasons for answering ‘Yes’ are:   1. Differences between intervention groups in the proportions of missing outcome data. If there is a difference between the effects of the experimental and comparator interventions on the outcome, and the missingness in the outcome is influenced by its true value, then the proportions of missing outcome data are likely to differ between intervention groups. Such a difference suggests a risk of bias due to missing outcome data, because the trial result will be sensitive to missingness in the outcome being related to its true value. For time-to-event-data, the analogue is that rates of censoring (loss to follow-up) differ between the intervention groups. 2. Reported reasons for missing outcome data provide evidence that missingness in the outcome depends on its true value; 3. Reported reasons for missing outcome data differ between the intervention groups; 4. The circumstances of the trial make it likely that missingness in the outcome depends on its true value. For example, in trials of interventions to treat schizophrenia it is widely understood that continuing symptoms make drop out more likely. 5. In time-to-event analyses, participants’ follow up is censored when they stop or change their assigned intervention, for example because of drug toxicity or, in cancer trials, when participants switch to second-line chemotherapy.   Answer ‘No’ if the analysis accounted for participant characteristics that are likely to explain the relationship between missingness in the outcome and its true value. | NA/Y/PY/PN/N/NI |
| **Risk-of-bias judgement** | See algorithm. | Low / High / Some concerns |
| Optional: What is the predicted direction of bias due to missing outcome data? | If the likely direction of bias can be predicted, it is helpful to state this. The direction might be characterized either as being towards (or away from) the null, or as being in favour of one of the interventions. | NA / Favours experimental / Favours comparator / Towards  null /Away from null /  Unpredictable |


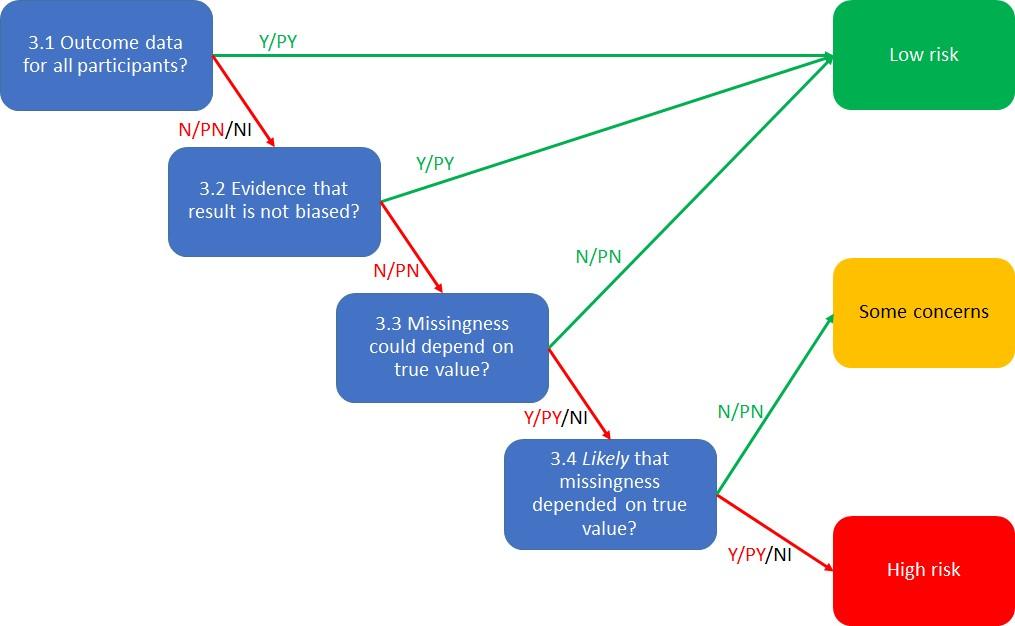


**Algorithm for suggested judgement of risk of bias due to missing outcome data**

## Domain 4: Risk of bias in measurement of the outcome

| **Signalling questions** | **Elaboration** | **Response options** |
| --- | --- | --- |
| **4.1 Was the method of measuring the outcome inappropriate?** | This question aims to identify methods of outcome measurement (data collection) that are unsuitable for the outcome they are intended to evaluate. The question *does not* aim to assess whether the choice of outcome being evaluated was sensible (e.g. because it is a surrogate or proxy for the main outcome of interest). In most circumstances, for pre-specified outcomes, the answer to this question will be ‘No’ or ‘Probably no’.  Answer ‘Yes’ or ‘Probably yes’ if the method of measuring the outcome is inappropriate, for example because:   1. it is unlikely to be sensitive to plausible intervention effects (e.g. important ranges of outcome values fall outside levels that are detectable using the measurement method); or 2. the measurement instrument has been demonstrated to have poor validity. | Y/PY/PN/N/NI |
| **4.2 Could measurement or ascertainment of the outcome have differed between intervention groups?** | Comparable methods of outcome measurement (data collection) involve the same measurement methods and thresholds, used at comparable time points. Differences between intervention groups may arise because of ‘diagnostic detection bias’ in the context of passive collection of outcome data, or if an intervention involves additional visits to a healthcare provider, leading to additional opportunities for outcome events to be identified. | Y/PY/PN/N/NI |
| **4.3 If N/PN/NI to 4.1 and 4.2: Were outcome assessors aware of the intervention received by study participants?** | Answer ‘No’ if outcome assessors were blinded to intervention status. For participant-reported outcomes, the outcome assessor is the study participant. | NA/Y/PY/PN/N/NI |
| **4.4 If Y/PY/NI to 4.3: Could assessment of the outcome have been influenced by knowledge of intervention received?** | Knowledge of the assigned intervention could influence participant-reported outcomes (such as level of pain), observer-reported outcomes involving some judgement, and intervention provider decision outcomes. They are unlikely to influence observer-reported outcomes that do not involve judgement, for example all-cause mortality. | NA/Y/PY/PN/N/NI |
| **4.5 If Y/PY/NI to 4.4: Is it likely that assessment of the outcome was influenced by knowledge of intervention received?** | This question distinguishes between situations in which (i) knowledge of intervention status could have influenced outcome assessment but there is no reason to believe that it did (assessed as ‘Some concerns’) from those in which (ii) knowledge of intervention status was likely to influence outcome assessment (assessed as ‘High’). When there are strong levels of belief in either beneficial or harmful effects of the intervention, it is more likely that the outcome was influenced by knowledge of the intervention received. Examples may include patient-reported symptoms in trials of homeopathy, or assessments of recovery of function by a physiotherapist who delivered the intervention. | NA/Y/PY/PN/N/NI |
| **Risk-of-bias judgement** | See algorithm. | Low / High / Some concerns |
| Optional: What is the predicted direction of bias in measurement of the outcome? | If the likely direction of bias can be predicted, it is helpful to state this. The direction might be characterized either as being towards (or away from) the null, or as being in favour of one of the interventions. | NA / Favours experimental / Favours comparator / Towards  null /Away from null /  Unpredictable |


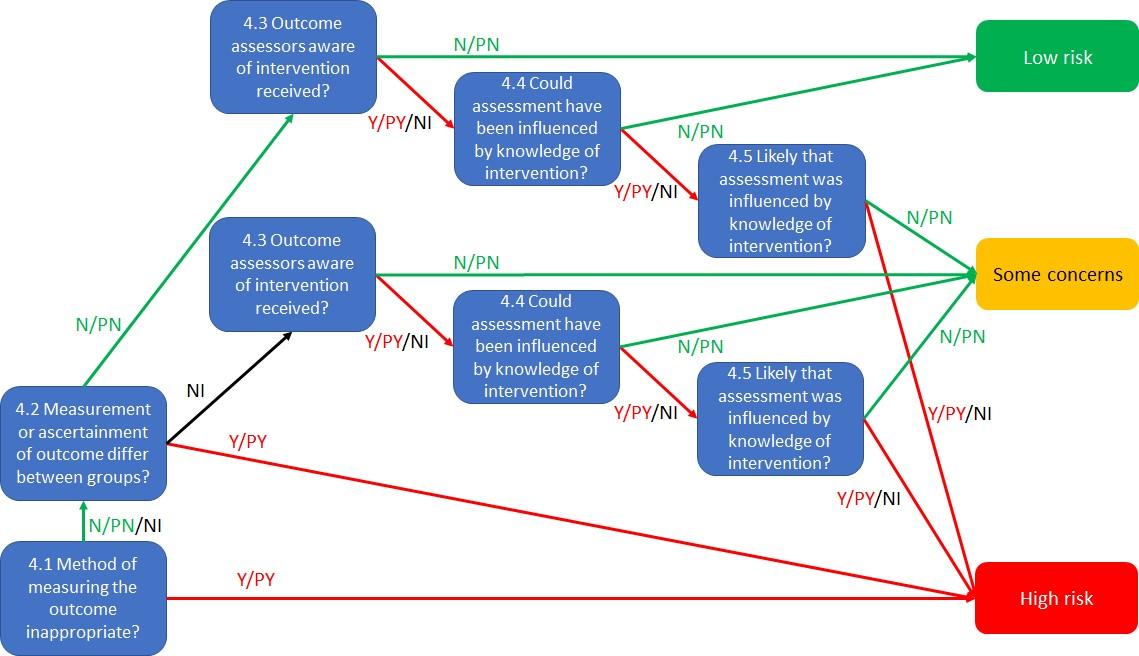


**Algorithm for suggested judgement of risk of bias in measurement of the outcome**

## Domain 5: Risk of bias in selection of the reported result

| **Signalling questions** | **Elaboration** | **Response options** |
| --- | --- | --- |
| **5.1 Were the data that produced this result analysed in accordance with a pre-specified analysis plan that was finalized before unblinded outcome data were available for analysis?** | If the researchers’ pre-specified intentions are available in sufficient detail, then planned outcome measurements and analyses can be compared with those presented in the published report(s). To avoid the possibility of selection of the reported result, finalization of the analysis intentions must precede availability of unblinded outcome data to the trial investigators.  Changes to analysis plans that were made before unblinded outcome data were available, or that were clearly unrelated to the results (e.g. due to a broken machine making data collection impossible) do not raise concerns about bias in selection of the reported result. | Y/PY/PN/N/NI |
| **Is the numerical result being assessed likely to have been selected, on the basis of the results, from...** |  |  |
| **5.2. ... multiple eligible**  **outcome measurements**  **(e.g. scales, definitions, time points) within the outcome domain?** | A particular outcome domain (i.e. a true state or endpoint of interest) may be **measured** in multiple ways. For example, the domain pain may be measured using multiple scales (e.g. a visual analogue scale and the McGill Pain Questionnaire), each at multiple time points (e.g. 3, 6 and 12 weeks posttreatment). If multiple measurements were made, but only one or a subset is reported on the basis of the results (e.g. statistical significance), there is a high risk of bias in the fully reported result. Attention should be restricted to outcome measurements that are eligible for consideration by the RoB 2 tool user. For example, if only a result using a specific measurement scale is eligible for inclusion in a meta-analysis (e.g. Hamilton Depression Rating Scale), and this is reported by the trial, then there would not be an issue of selection even if this result was reported (on the basis of the results) in preference to the result from a different measurement scale (e.g. Beck Depression Inventory).  Answer ‘Yes’ or ‘Probably yes’ if:  There is clear evidence (usually through examination of a trial protocol or statistical analysis plan) that a domain was measured in multiple eligible ways, but data for only one or a subset of measures is fully reported (without justification), and the fully reported result is likely to have been selected on the basis of the results. Selection on the basis of the results can arise from a desire for findings to be newsworthy, sufficiently noteworthy to merit publication, or to confirm a prior hypothesis. For example, trialists who have a preconception, or vested interest in showing, that an | Y/PY/PN/N/NI |

|  | experimental intervention is beneficial may be inclined to report outcome measurements selectively that are favourable to the experimental intervention.  Answer ‘No’ or ‘Probably no’ if:  There is clear evidence (usually through examination of a trial protocol or statistical analysis plan) that all eligible reported results for the outcome domain correspond to all intended outcome measurements.  or  There is only one possible way in which the outcome domain can be measured (hence there is no opportunity to select from multiple measures). or  Outcome measurements are inconsistent across different reports on the same trial, but the trialists have provided the reason for the inconsistency and it is not related to the nature of the results.  Answer ‘No information’ if:  Analysis intentions are not available, or the analysis intentions are not reported in sufficient detail to enable an assessment, and there is more than one way in which the outcome domain could have been measured. |  |
| --- | --- | --- |
| **5.3 ... multiple eligible analyses of the data?** | A particular outcome measurement may be analysed in multiple ways. Examples include: unadjusted and adjusted models; final value vs change from baseline vs analysis of covariance; transformations of variables; different definitions of composite outcomes (e.g. ‘major adverse event’); conversion of continuously scaled outcome to categorical data with different cut-points; different sets of covariates for adjustment; and different strategies for dealing with missing data. Application of multiple methods generates multiple effect estimates for a specific outcome measurement. If multiple estimates are generated but only one or a subset is reported on the basis of the results (e.g. statistical significance), there is a high risk of bias in the fully reported result. Attention should be restricted to analyses that are eligible for consideration by the RoB 2 tool user. For example, if only the result from an analysis of post-intervention values is eligible for inclusion in a meta-analysis (e.g. at 12 weeks after randomization), and this is reported by the trial, then there would not be an issue of selection even if this result was reported (on the basis of the results) in preference to the result from an analysis of changes from baseline.  Answer ‘Yes’ or ‘Probably yes’ if: | Y/PY/PN/N/NI |
|  | There is clear evidence (usually through examination of a trial protocol or statistical analysis plan) that a measurement was analysed in multiple eligible ways, but data for only one or a subset of analyses is fully reported (without justification), and the fully reported result is likely to have been selected on the basis of the results. Selection on the basis of the results arises from a desire for findings to be newsworthy, sufficiently noteworthy to merit publication, or to confirm a prior hypothesis. For example, trialists who have a preconception or vested interest in showing that an experimental intervention is beneficial may be inclined to selectively report analyses that are favourable to the experimental intervention.  Answer ‘No’ or ‘Probably no’ if:  There is clear evidence (usually through examination of a trial protocol or statistical analysis plan) that all eligible reported results for the outcome measurement correspond to all intended analyses.  or  There is only one possible way in which the outcome measurement can be analysed (hence there is no opportunity to select from multiple analyses).  or  Analyses are inconsistent across different reports on the same trial, but the trialists have provided the reason for the inconsistency and it is not related to the nature of the results.  Answer ‘No information’ if:  Analysis intentions are not available, or the analysis intentions are not reported in sufficient detail to enable an assessment, and there is more than one way in which the outcome measurement could have been analysed. |  |
| **Risk-of-bias judgement** | See algorithm. | Low / High / Some concerns |
| Optional: What is the predicted direction of bias due to selection of the reported result? | If the likely direction of bias can be predicted, it is helpful to state this. The direction might be characterized either as being towards (or away from) the null, or as being in favour of one of the interventions. | NA / Favours experimental / Favours comparator / Towards  null /Away from null /  Unpredictable |


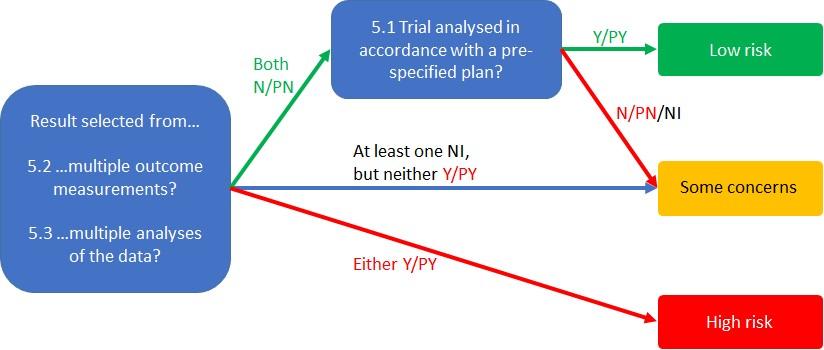


**Algorithm for suggested judgement of risk of bias in selection of the reported result**

Overall risk of bias

| **Risk-of-bias judgement** |  | Low / High / Some concerns |
| --- | --- | --- |
| Optional: What is the overall predicted direction of bias for this outcome? |  | Favours experimental /  Favours comparator / Towards null /Away from null / Unpredictable / NA |

| **Overall risk-of-bias judgement** | **Criteria** |
| --- | --- |
| Low risk of bias | The study is judged to be at **low risk of bias** **for all domains** for this result. |
| Some concerns | The study is judged to raise **some concerns** in at least one domain for this result, but not to be at high risk of bias for any domain. |
| High risk of bias | The study is judged to be at **high risk of bias** in at least one domain for this result.  Or  The study is judged to have **some concerns** for **multiple domains** in a way that substantially lowers confidence in the result. |

The development of the RoB 2 tool was supported by the MRC Network of Hubs for Trials Methodology Research (MR/L004933/2- N61), with the support of the host MRC ConDuCT-II Hub (Collaboration and innovation for Difficult and Complex randomised controlled Trials In Invasive procedures - MR/K025643/1), by MRC research grant MR/M025209/1, and by a grant from The Cochrane Collaboration.


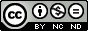


This work is licensed under a [Creative Commons Attribution-NonCommercial-NoDerivatives 4.0 International License.](http://creativecommons.org/licenses/by-nc-nd/4.0/)

# Standards of Reporting for MRI-targeted Biopsy Studies (START) of the Prostate

| In studies of MRI-targeted biopsies, the following items should be reported: | Page.No |
| --- | --- |
| Title and Introduction | . |
| 1 Identification as a study reporting results from MRI-targeted biopsy of the prostate, with a clear statement of the study aim | . |
| Study methodology | . |
| 2 The study design (eg, prospective or retrospective, cohort or randomized) | . |
| 3 The dates of recruitment, including whether any men have been included in previously published cohorts | . |
| 4 Whether recruitment was based on PSA values alone or results from other tests, such as MRI, TRUS, or biopsy | . |
| Study population | . |
| 5 The biopsy and treatment status of the population, specifying: | . |
| a. Number of men without prior biopsy | . |
| b. Number of men with prior biopsy negative for cancer | . |
| c. Number of men with prior biopsy positive for cancer and the number of men in each Gleason score category (eg, 3 + 3, 3 + 4, 4 + 3, 4 + 4) | . |
| d. Number of men with previous treatment to the prostate | . |
| 6 Summary measures (range and mean or median) for age, prostate volume, and prebiopsy PSA | . |
| 7 A flow chart of the number of men who were suitable for study inclusion, those who were then excluded (with reasons specified), and those who completed the study | . |
| Conduct and reporting of the MRI | . |
| 8 The field strength of the magnet, specific coils used (eg, pelvic, endorectal), and a brief description of the sequences, including: | . |
| a. Slice thickness and true acquisition resolution based on the field of view and reconstruction matrix for T2-weighted imaging, DWI, and DCE | . |
| b. For T2-weighted imaging, which planes were acquired | . |
| c. For DCE, the temporal resolution and the model used for postprocessing | . |
| d. For DWI, the b-values used, which image sets were analysed (eg, high b-value image, ADC map, or both) and whether qualitative or quantitative analysis was carried out | . |
| 9 The reporting method used, including the use of any scoring system for suspicion of prostate cancer, whether a prose or diagrammatic report was used, and whether the radiologist was blinded to the clinical information | . |
| 10 The experience of the reporting radiologist (eg, number of years) | . |
| Conduct of the biopsy | . |
| 11 Whether targeted cores or standard cores were taken first, whether they were potted separately, and the approach used for each technique (eg, transrectal, transperineal) | . |
| 12 The method of registration and guidance used for MRI-targeted biopsy, including: | . |
| a. The type of registration used (eg, visual or software registration) | . |
| b. For visual registration, whether the biopsy operator had direct access to the MRI images or used a prose or diagrammatic report | . |
| c. For software registration, the software name and version, the MRI-sequence used for registration, and whether registration was rigid or non-rigid | . |
| d. The guidance used during the biopsy procedure (eg, ultrasound or MRI) | . |
| 13 Whether the person taking the standard cores was aware of the location of the lesion on MRI | . |
| Results | . |
| 14 The number of men who had an MRI with a suspicious lesion and the number who had an MRI-targeted biopsy | . |
| 15 A summary measure (mean or median) of the number of targeted cores taken per prostate or per lesion and of the number of standard cores taken per prostate | . |
| 16 The number of men in each Gleason score category (eg, 3 + 3, 3 + 4, 4 + 3, 4 + 4) from targeted cores alone and standard cores alone | . |
| 17 The number of men with clinically significant and clinically insignificant cancer detected by standard cores alone and targeted cores alone, with the criteria used for the definition of clinically significance | . |
| 18 A cross-tabulation of the number of men with clinically significant and clinically insignificant cancer detected by targeted biopsies against the number detected by standard biopsies | . |
| 19 The proportion of cores positive for clinically significant cancer in targeted cores alone and standard cores alone and the mean number of cores taken per diagnosis of clinically significant cancer for each technique | . |
| Discussion | . |
| 20 A comparison between targeted and standard biopsy techniques for: | . |
| a. Proportion of cores positive for clinically significant cancer | . |
| b. Sampling efficiency (eg, mean number of cores taken per diagnosis of clinically significant cancer) | . |
| c. Number of men diagnosed with clinically significant and clinically insignificant cancer | . |
| MRI = magnetic resonance imaging; PSA = prostate-specific antigen, TRUS = transrectal ultrasound; DWI = diffusion-weighted imaging; DCE = dynamic contrast-enhanced imaging, ADC = apparent diffusion coefficient. | . |

# Intention-to-treat and modified intention-to-treat definitions and approaches

The intention-to-treat sample is defined as shown in the table below.


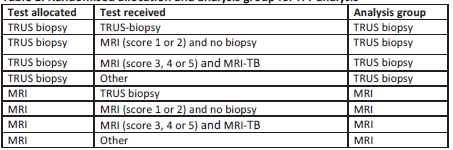


If a man received the allocated test as well as another test, only the results of the allocated test will be use for the ITT analysis as shown in the table below.


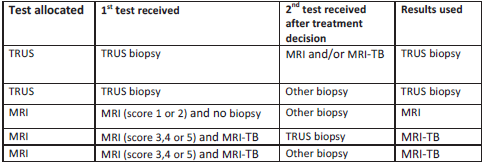


The table below shows comparisons of ITT, modified ITT (complete case analysis) and per protocol analyses for TRUS Biopsy arm and the conditions for excluding patients based on potential protocol deviations from the protocol.


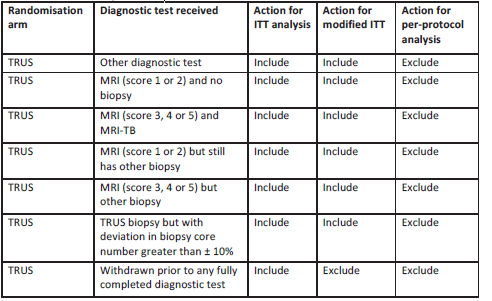


The table below shows comparisons of ITT, modified ITT (complete case analysis) and per protocol analyses for MRI-targeted biopsy arm and the conditions for excluding patients based on potential protocol deviations from the protocol.


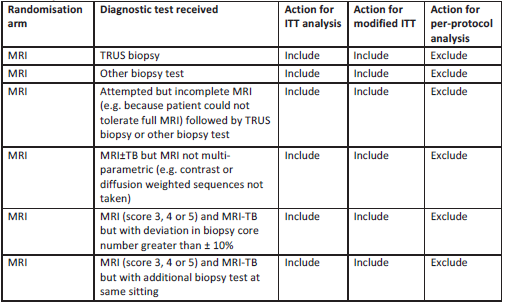

Supplement: S1 Appendix — (DOCX) [file pone.0263345.s001.docx]
